# Single cell lineage analysis of mouse embryonic stem cells at the exit from pluripotency

**DOI:** 10.1242/bio.20135934

**Published:** 2013-08-19

**Authors:** Jamie Trott, Alfonso Martinez Arias

**Affiliations:** 1 Department of Genetics, University of Cambridge, Cambridge CB2 3EH, UK; 2 Wellcome Trust Centre for Stem Cell Research, University of Cambridge, Cambridge CB2 1QR, UK; * Present address: Institute of Medical Biology, 8A Biomedical Grove, No. 06-06 Immunos, Singapore 138648

**Keywords:** Single cell analysis, Pluripotency, Lineage priming, Mouse embryonic stem cells, Transcription factor networks, Wnt/β-catenin signalling

## Abstract

Understanding how interactions between extracellular signalling pathways and transcription factor networks influence cellular decision making will be crucial for understanding mammalian embryogenesis and for generating specialised cell types in vitro. To this end, pluripotent mouse Embryonic Stem (mES) cells have proven to be a useful model system. However, understanding how transcription factors and signalling pathways affect decisions made by individual cells is confounded by the fact that measurements are generally made on groups of cells, whilst individual mES cells differentiate at different rates and towards different lineages, even in conditions that favour a particular lineage. Here we have used single-cell measurements of transcription factor expression and Wnt/β-catenin signalling activity to investigate their effects on lineage commitment decisions made by individual cells. We find that pluripotent mES cells exhibit differing degrees of heterogeneity in their expression of important regulators from pluripotency, depending on the signalling environment to which they are exposed. As mES cells differentiate, downregulation of *Nanog* and *Oct4* primes cells for neural commitment, whilst loss of *Sox2* expression primes cells for primitive streak commitment. Furthermore, we find that Wnt signalling acts through *Nanog* to direct cells towards a primitive streak fate, but that transcriptionally active β-catenin is associated with both neural and primitive streak commitment. These observations confirm and extend previous suggestions that pluripotency genes influence lineage commitment and demonstrate how their dynamic expression affects the direction of lineage commitment, whilst illustrating two ways in which the Wnt signalling pathway acts on this network during cell fate assignment.

## Introduction

The mammalian embryo is derived from a small group of cells, the epiblast, which is first segregated from extraembryonic tissue just before implantation, at the blastocyst stage (Tam and Loebel, 2007; Rossant and Tam, 2009). This group of cells forms an epithelium (the postimplantation epiblast) that undergoes a proliferative expansion before giving rise to different lineages through the spatio-temporal segregation of patterns of gene expression. The first symmetry-breaking event in the embryonic epiblast occurs around the time of implantation (E4.5), with the definition of an anteroposterior axis, mediated by signalling between the extraembryonic endoderm and the epiblast (Takaoka et al., 2006). This event positions the primordium for the brain (ectoderm) anteriorly, and the primitive streak, that will give rise to the endoderm and the mesoderm, posteriorly (Kimura et al., 2000; Perea-Gomez et al., 2002; Yamamoto et al., 2004; Rivera-Pérez and Magnuson, 2005; Arkell and Tam, 2012). The transition from the blastocyst to the patterned epiblast is a continuous process that preludes the diversification and spatial organization of the embryo and the signalling pathways involved have been studied in detail (Tam and Loebel, 2007). However, the molecular events that initially specify a cell as anterior or posterior in the epiblast, have not been identified, as they coincide with implantation of the embryo. Embryonic Stem (ES) cells provide an alternative and complementary system to study these events.

Mouse Embryonic Stem (mES) cells are clonal populations of cultured cells derived from the blastocyst of the embryo that can give rise to all of the cell types that constitute the adult organism, i.e. they are pluripotent (Bradley et al., 1984; Smith, 2001). Several studies have revealed that pluripotent cells in vitro fluctuate between a ground state of pluripotency and one in which cells are primed for lineage commitment (Chambers et al., 2007; Hayashi et al., 2008; Toyooka et al., 2008; Kalmar et al., 2009; Canham et al., 2010; Martinez Arias and Brickman, 2011). Manipulation of different extracellular signalling pathways can shift the population as a whole towards ground state pluripotency or cause germ layer differentiation, depending on the combination of signals the cells are exposed to (Ying et al., 2008; Wray et al., 2010). Leukemia Inhibitory Factor (LIF) and Bone Morphogenetic Protein (BMP) are sufficient for self-renewal of pluripotency of an mES cell population in a chemically defined medium like N2B27 (Ying et al., 2003a). However, culturing cells in the presence of inhibitors of Extracellular-signal-Regulated Kinases (ERK) and Glycogen Synthase Kinase 3 (GSK3), hereafter referred to as “2i” conditions, shifts cells to ground state pluripotency (Ying et al., 2008). Conversely, in N2B27 basal medium alone, which contains the Retinoic Acid precursor Vitamin A and insulin, pluripotent cells adopt a neural fate, whilst activation of Wnt or Nodal signalling elicits a primitive streak like population that serves as a precursor for the endoderm and the mesoderm (Ying et al., 2003b; Gadue et al., 2006; Bakre et al., 2007; Nostro et al., 2008; Hansson et al., 2009). On the assumption that mES cells reflect an embryonic population, they can be used as a model to study cell fate assignment and maintenance as well as the early stages of mammalian development.

Here we have analyzed the controlled exit of mES cells from pluripotency under established neural differentiation conditions (N2B27) by measuring gene expression in single cells. This technique has previously been used to study mouse blastocysts (Guo et al., 2010), pluripotent mES cells (Hayashi et al., 2008; Tang et al., 2010; MacArthur et al., 2012; Trott et al., 2012) and haematopoietic stem cells (Pina et al., 2012; Moignard et al., 2013), but our study represents the first analysis of the early stages of differentiation of mES cells.

We find that initial differentiation of mES cells in N2B27 is not restricted to neural lineages, but that during the first four days of differentiation, individual cells express markers for a variety of lineages. Furthermore, our single cell analysis suggests that the expression of individual components of the core pluripotency network (*Nanog*, *Oct4* and *Sox2*) restricts differentiation to specific lineages as cells exit pluripotency. Our findings lead us to suggest that during the first four days of differentiation in culture, cells undergo a transition that mimics events in vivo between the blastocyst and the postimplantation epiblast, by the end of which cells are biased towards commitment to different lineages. We also find that, during this period, ES cells display a widespread activation of canonical Wnt/β-catenin signalling which is not affiliated with specific lineages and discuss the possible implications of this observation.

## Results

### Pluripotency at the single cell level

Mouse ES cells self-renew under a variety of culture conditions and attempts to reveal key molecules supporting pluripotency revealed that LIF is central in the promotion of “naïve pluripotency” (Smith et al., 1988; Williams et al., 1988). Subsequently it was found that mES cells placed in synthetic medium (N2B27) in the presence of LIF and BMP4 maintained their pluripotency, which demonstrated that BMP is the component in Serum responsible for inhibition of differentiation (Ying et al., 2003a). A number of experiments have since established that heterogeneity in the expression of *Rex1*, *Nanog*, *Klf4* and *Stella* is a hallmark of naïve pluripotent mES cells, with cells expressing high levels being pluripotent and cells expressing low levels being primed for differentiation, as reflected by their increased propensity to exit pluripotency permanently (Chambers et al., 2007; Hayashi et al., 2008; Toyooka et al., 2008; Kalmar et al., 2009). Furthermore, in Serum and LIF or LIF and BMP, cells can transit between different expression states. Culturing mES cells in 2i conditions eliminates differentiation-primed cells from the culture and leads to a robust state of pluripotency that has been termed “ground state pluripotency” and that can be propagated in these growth conditions (Ying et al., 2008; Wray et al., 2010). Accordingly, culturing cells in 2i increases colony formation and chimaera contribution rates.

**Fig. 1.**
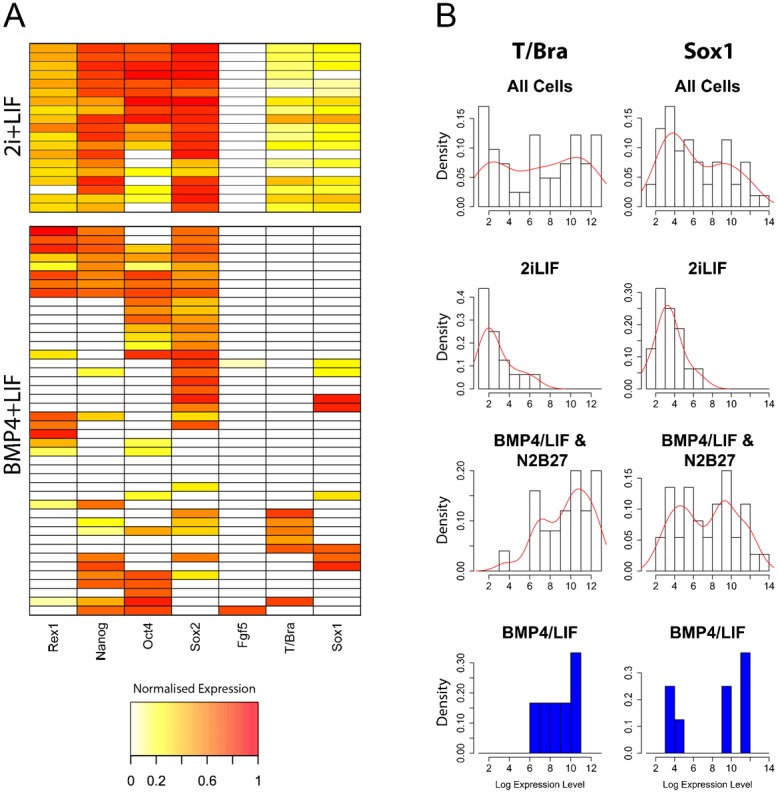
Gene expression in individual wild-type (E14Tg2A) pluripotent mES cells in N2B27 supplemented with BMP4+LIF or 2i+LIF. (A) Heat maps of pluripotency (*Rex1*, *Nanog*, *Oct4* and *Sox2*) and differentiation (*Fgf5, T/Bra and Sox1*) marker gene expression in mES cells maintained in 2i+LIF or BMP4+LIF. Plotted values are natural logarithms of *Gapdh*-normalised expression levels, scaled such that cells with no expression of a particular gene have a value of 0 whilst cells that express the maximum detected level of a gene have a value of 1. (B) Histograms of primitive streak (*T/Bra*) and neural (*Sox1*) marker gene expression in different groups of mES cells. Natural logarithms of *Gapdh*-normalised expression levels are plotted as histograms (black outlined bars) with the corresponding probability density functions overlaid (red lines). The top row of histograms show data for all cells cultured in 2i+LIF, BMP4+LIF and after one to four days BMP4+LIF withdrawal (see Materials and Methods), whilst the lower three rows show data for subsets of the above. Only cells with detectable expression of the gene in question are included. The histograms show bimodal expression level distributions for both *Sox1* and *T/Bra*, suggesting there are discrete groups of cells expressing high or low levels of these genes. These differentiation markers are only ever expressed at low levels by cells cultured in 2i+LIF, and are often coexpressed in individual cells (Fig. 1A). Conversely, in BMP4+LIF and after BMP4+LIF withdrawal (N2B27), these genes can be expressed at high or low levels, but tend not to be coexpressed (Fig. 1A). These observations suggest that cells expressing high levels of these genes are differentiating, whilst low-level expression of these genes is not indicative of commitment to a particular lineage. The fact that some cells in BMP4+LIF express high levels of *Sox1* or *T/Bra* suggests these populations contain a proportion of differentiating cells.

To determine whether the enhanced pluripotency of mES cells cultured in 2i has a transcriptional basis at the level of single cells, expression of several key markers of pluripotency and lineage commitment was measured directly in individual cells by qPCR (Fig. 1A). In the presence of LIF and BMP4, it is possible to observe some cells expressing differentiation markers: *Sox1* (neural) and *T/Bra* (Primitive Streak, the precursor of the mesoderm and the endoderm) (Herrmann et al., 1990; Wood and Episkopou, 1999; Smith, 2004). These cells express low levels of pluripotency markers and are likely to represent the differentiating population known to be present in these conditions. When cells are placed in 2i (in this case supplemented with LIF), the heterogeneities disappear and ∼80% of the cells express high and homogeneous levels of *Oct4*, *Sox2* and *Nanog* alongside negligible levels of differentiation markers (Fig. 1B). This demonstrates that the enhanced pluripotency of cells cultured in 2i is associated with more stable expression of key pluripotency regulators at the level of single cells and consequently a lack of differentiation in such cultures.

### The exit from the pluripotent state

**Fig. 2.**
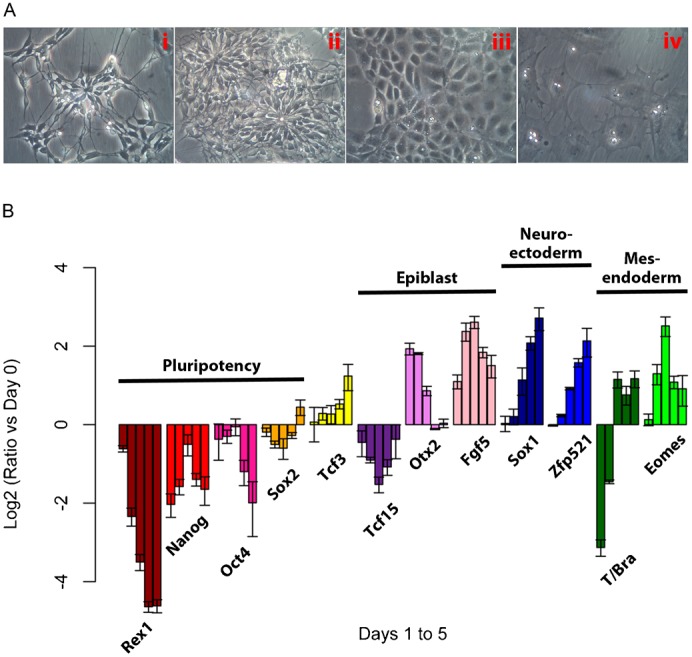
Changes in morphology and gene expression as mES cells are differentiated over five days in N2B27 medium following BMP4+LIF withdrawal (see Materials and Methods). (A) Examples of cellular morphologies observed after five days in N2B27: neural rosettes (i,ii), epithelial (iii) and mesenchymal (iv). (B) Population level changes in gene expression over the course of five days of differentiation. Expression of markers of pluripotency (*Rex1*, *Nanog*, *Oct4* and *Sox2*), epiblast (*Tcf15*, *Otx2* and *Fgf5*), neuroectoderm (*Sox1* and *Zfp521*) and mesendoderm (*T/Bra* and *Eomes*) was measured. For each gene on each of the days from day 1 (left most bar) to day 5 (right most bar), the value plotted is the log_2_ of the ratio on that day compared to the level in BMP4+LIF (both values normalised to the levels of the gene *Ppia*). Error bars above and below each bar represent the standard deviation of the mean of 3 or more qPCR replicates.

Removal of LIF and BMP4 from an N2B27 based culture medium is often used to trigger loss of pluripotency and the differentiation of the culture towards neural lineages (Ying et al., 2003b); in these conditions, addition of Retinoic Acid (RA) is used to enhance this effect and we have observed that it accelerates differentiation, though it increases cell death (J.T. and A.M.A., unpublished observations). As mES cells begin to differentiate, they undergo two sequential transitions: firstly from a multi-layered to a monolayer epithelium – which presumably reflects the transition to an EpiSC state – and secondly, after five or six days, through an Epithelial Mesenchymal Transition (EMT), to a phenotypically heterogeneous population. While some cells form rosette like structures typical of neural cells, others have morphologies typical of mesenchymal or epithelial tissues (Fig. 2A). Prolonged culture in N2B27 appears to eliminate the latter and allows the expansion of a population with neural characteristics, such that by six days cells express high levels of *Sox1* (Ying et al., 2003b; Abranches et al., 2009; Engberg et al., 2010; Stavridis et al., 2010). These observations raised the possibility that N2B27 might not provide an environment to direct differentiation strictly into neural fates during the exit from pluripotency and led us to monitor gene expression across different lineages as cells exit pluripotency in N2B27.

Cells that had been grown in the presence of LIF and BMP4 were placed in N2B27 and the expression of several genes, some associated with pluripotency and some with differentiation, was monitored daily over time (Fig. 2B). We observed the expected rapid decrease in *Rex1*, *Nanog* and *Oct4* expression as cells exit pluripotency, as well as transient expression of the epiblast affiliated genes *Fgf5* and *Otx2*. There is also an expected, steady increase in the expression of genes associated with neural fates (*Sox1* and *Zfp521*) and an early decrease in the expression of *Sox2* as cells exit pluripotency, followed by an increase as cells adopt neural fates. However, we also observed expression of *T/Bra* and *Eomes*, which are genes associated with Primitive Streak and endodermal fates (the sharp decline in *T/Bra* levels on day 1 most likely represents the failure of cells expressing this gene in BMP4+LIF conditions to re-adhere when reseeded). These profiles additionally reveal a small, but significant and reproducible, rise in the levels of *Nanog* and *Oct4* on day 3 of differentiation, which can be observed, but is rarely remarked on, in many reports. Dynamic expression of *Nanog* and *Oct4* probably reflects reactivation of these genes as cells pass through the epiblast state, where both are expressed (Brons et al., 2007). These results demonstrate that the early stages of differentiation in N2B27 involve expression of fates other than neural commitment.

### The exit from pluripotency from the perspective of the pluripotency network

Population analyses, like the one described above, provide a useful guide to the behaviour of an ensemble of cells, but are less helpful when trying to determine how individual cells choose between different lineages during differentiation. Therefore, we used single cell qPCR to identify changes in gene expression that occur as cells exit pluripotency. Individual cells were selected at random from mES cell populations over four days of differentiation in N2B27 and processed for measuring gene expression. We were able to obtain 19 cells grown in 2i+LIF, 45 cells grown in LIF and BMP4, and 44, 42, 36 and 34 cells for each of one, two, three and four days following withdrawal of LIF and BMP4 (the cells were taken from the same populations for which bulk gene expression was presented in Fig. 2B). Expression of genes representative of pluripotency (*Rex1*, *Nanog*, *Oct4* and *Sox2*), epiblast differentiation (*Fgf5*), neural commitment (*Sox2* and *Sox1*) and primitive streak commitment (*T/Bra*) was measured to determine the differentiation state of individual cells over the course of the experiment (for details of the procedure, see Materials and Methods).

**Fig. 3.**
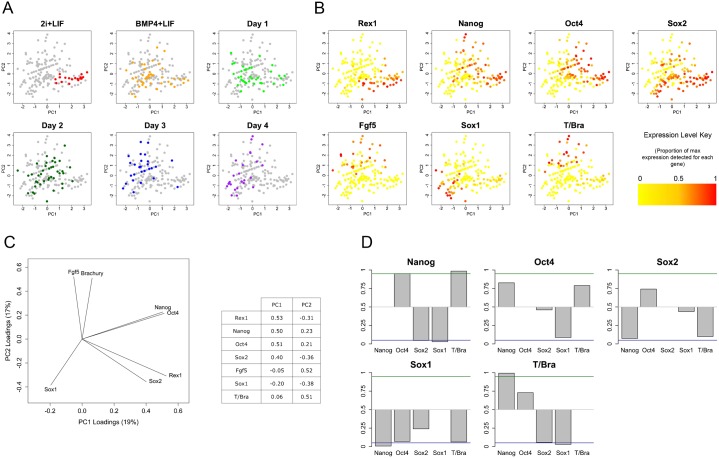
Relationships between the expression levels of markers of pluripotency and lineage commitment in mES cells exiting pluripotency. The expression of markers of pluripotency (*Rex1*, *Nanog*, *Oct4* and *Sox2*) and lineage commitment (*Fgf5*, *Sox1* and *T/Bra*) was measured in mES cells maintained in 2i+LIF, BMP4+LIF or differentiated over four days in N2B27 following withdrawal of BMP4 and LIF. Natural logarithms of *Gapdh*-normalised expression levels were used for all calculations relating to this figure and for plotting. (A,B,C) Principal Components Analysis (PCA) of gene expression in individual mES cells. Each point in the plots in panels A and B represents a single cell, the position of which is a function of the expression of seven genes (C) in that cell. The positions of individual cells are identical in each of the plots shown. The cells analysed here, with the exception of those cultured in 2i+LIF, were taken from the same population as those used to produce the population level gene expression data in Fig. 2. Individual cells are coloured according to treatment/differentiation day (A) or their expression of a particular gene (B). In panel B the natural logarithms of the *Gapdh*-normalised expression levels (including cells in which expression was not detected) are scaled such that they range between 0 and 1. Colours are then mapped to these values as shown by the colour key. The principal component loading values used to calculate the positions of individual cells and the percentage of the variability in the dataset that is explained by each axis are shown in panel C. (D) Relationships between the expression of pluripotency and differentiation genes after three to four days differentiation in N2B27. Cells were segregated (in silico) according to whether or not they express a first gene (plot titles). The mean level of a second gene (horizontal labels below each bar) in cells that express the first gene was then calculated. Bootstrapping was used to determine the probability that the mean level of the second gene was higher in cells that express the first gene than would be expected by chance (*P* value). These *P* values were plotted as grey bars. Bootstrapping involves shuffling the levels of the second gene across the population and repeating the calculation described above 100,000 times; the *P* value is the proportion of the bootstrap means that are lower than the real mean. A high *P* value indicates that the levels of two genes are positively correlated, whilst a low *P* values indicates they are negatively correlated. The green and blue lines at *P* values of 0.95 and 0.05 denote statistically significant relationships.

Principal Components Analysis (PCA) of the gene expression profiles of individual cells shows that in 2i conditions cells have very similar expression profiles, which gradually diverge as they differentiate (Fig. 3A). In 2i, cells cluster at the bottom right of the PCA plot alongside cells from other conditions that also express *Rex1* (Fig. 3B), indicating that cells in this region of the plot are pluripotent. After four days differentiation, most cells are to be found towards the top- or bottom-left of the plot where cells that express the primitive streak marker *T/Bra* or the neural marker *Sox1* are found. On days 1 and 2 following BMP4+LIF withdrawal, cells are dispersed between these two extremes, which suggests that, in vitro, individual cells proceed towards lineage commitment at different rates. Furthermore, coexpression of significant levels of *Brachyury* and *Sox1* is observed very rarely suggesting multi-lineage priming does not occur frequently in this system.

Patterns of pluripotency gene expression across the plot suggest that individual pluripotency genes influence the direction of lineage commitment (Fig. 3B,C). Pluripotent cells at the bottom right of the plot coexpress *Nanog*, *Oct4* and *Sox2* homogeneously. However, differentiating cells express different combinations of these genes, suggesting that the sequence in which the expression of pluripotency genes is downregulated differs between differentiating cells. Interestingly, cells that retain high levels of *Sox2* expression often coexpress *Sox1*, but very rarely express *T/Bra*, whilst the opposite is true of cells that retain high levels of *Nanog* and/or *Oct4* expression. These observations suggest that retention of *Sox2* expression predisposes cells towards neural commitment, whilst retention of *Nanog* or *Oct4* expression biases cells towards primitive streak commitment.

To test these relationships more formally we looked at probabilities of coexpression after four days differentiation (Fig. 3D). This analysis confirmed the strong anti-correlation between expression of *Nanog* and *T/Bra* on the one hand, and *Sox2* and *Sox1* on the other, in differentiating cells. The relationships between expression of *Oct4* and the other genes were similar to those of *Nanog*, but generally much weaker. These findings confirm earlier suggestions that different pluripotency genes specifically inhibit commitment to individual lineages, and additionally demonstrate how these interactions are relieved over time in order to guide cells towards different lineages.

### β-catenin biases lineage commitment through the regulation of *Nanog*

**Fig. 4.**
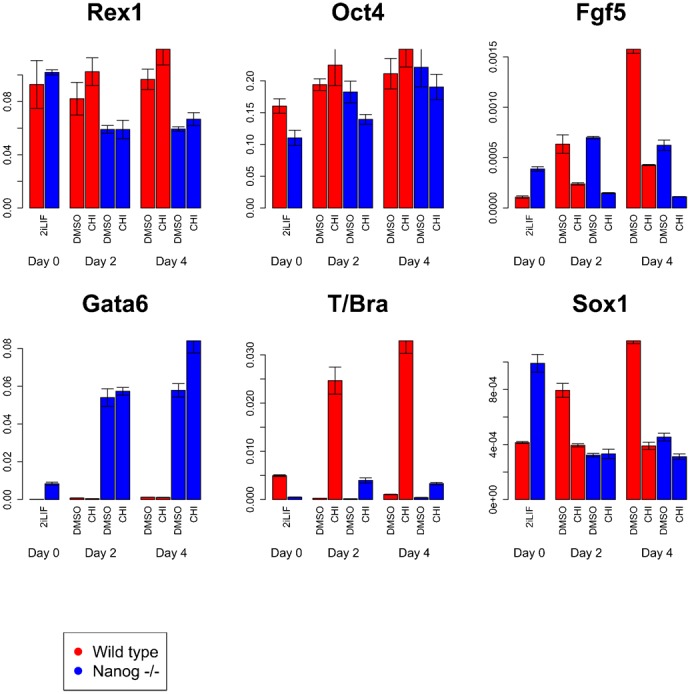
The effects of β-catenin stimulation on pluripotency and lineage marker gene expression in wild-type (E14Tg2A) and *Nanog−/−* (44Cre6) cells. Wild-type (red bars) and *Nanog*−/− (blue bars) mES cells maintained in 2i+LIF on gelatin-coated tissue culture plastic were transferred to serum+LIF to allow a proportion of cells to exit pluripotency (a percentage of mES cells differentiate spontaneously in serum+LIF), in the presence or absence of the β-catenin agonist Chiron. The effects of Chiron stimulation of β-catenin on expression of markers of pluripotency (*Rex1*, *Nanog* and *Oct4*), epiblast (*Fgf5*), neuroectoderm (*Sox1* and *Zfp521*), mesendoderm (*T/Bra*) and primitive endoderm (*Gata6*) were then assessed by qPCR after two and four days. Plotted are the levels of individual genes in each sample relative to the levels of the reference gene *Gapdh*. Error bars above and below each bar represent the standard deviation of the mean of at least 3 qPCR replicates. DMSO is used a control in this experiment as Chiron is dissolved in DMSO.

*Nanog* acts as a gatekeeper for pluripotency but, as we have shown here and others have observed previously, it is also associated with the emergence of a primitive streak fate (Hart et al., 2004; Chambers et al., 2007; Osorno et al., 2012). Similarly, β-catenin stimulation has been shown to promote both pluripotency and primitive streak commitment (Liu et al., 1999; Huelsken et al., 2000; Wray et al., 2011; Yi et al., 2011). Since β-catenin has been shown to enhance *Nanog* expression (Pereira et al., 2006; Yi et al., 2008), we tested whether β-catenin regulates pluripotency and lineage commitment via upregulation of *Nanog* expression by assessing the effects of activating Wnt/β-catenin signalling in the absence of *Nanog* (Fig. 4). *Nanog* mutant mES cells exhibit an unstable pluripotency as shown by their high rates of differentiation. However, Chiron, which activates β-catenin signalling, is able to block upregulation of *Fgf5* expression in the absence of *Nanog*, demonstrating that Chiron maintains pluripotency independently of *Nanog*.

We notice that *Nanog* mutant cells have a strong tendency to upregulate expression of the endoderm marker *Gata6*, which most likely reflects increased primitive endoderm commitment, consistent with the established role of *Nanog* in blocking primitive endoderm commitment (Mitsui et al., 2003). Nonetheless, *Nanog* mutant cells are able to commit to both neural and primitive streak fates (Chambers et al., 2007). However, we observe that in the absence of *Nanog*, β-catenin is unable to block *Sox1* expression or stimulate *T/Bra* expression significantly. These observations indicate that the ability of β-catenin to predispose cells towards primitive streak commitment is dependent on *Nanog*.

### Wnt signalling as a signature for the exit from pluripotency

**Fig. 5.**
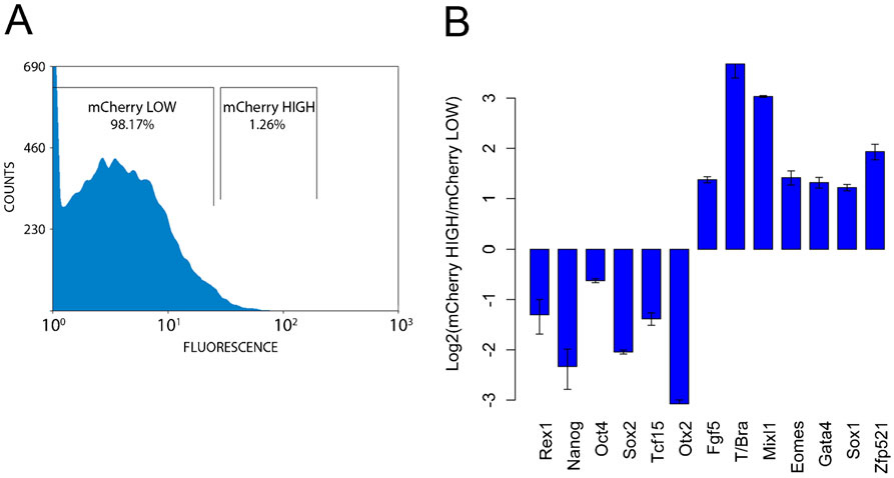
Wnt signalling marks the exit from pluripotency. (A) Fluorescence profile of mES cells cultured in serum+LIF that express an mCherry reporter of transcriptionally active β-catenin (Ferrer-Vaquer et al., 2010). The proportion of cells in each of the gates is shown. (B) Gene expression in cells sorted for expression of mCherry using the gates shown in panel A. The two populations were assessed for their expression of markers of pluripotency (*Rex1*, *Nanog*, *Oct4* and *Sox2*), epiblast differentiation (*Fgf5*), primitive streak and its derivatives (*T/Bra*, *Mixl1*, *Eomes* and *Gata4*) and neural differentiation (*Sox1* and *Zfp521*) using qPCR. Each bar shows the log_2_ value of the ratio of gene expression in mCherry high cells versus mCherry low cells. All values were first normalised to *Ppia* expression. Error bars above and below each bar represent the standard deviation of the mean of 3 or more qPCR replicates. *Axin2*, a direct transcriptional target of β-catenin signalling, was also measured in both populations and, as expected, was only expressed in the mCherry high population. Increased levels of β-catenin-mediated transcription are associated with loss of pluripotency gene expression and upregulation of markers of multiple opposing lineages.

When LIF is substituted for Retinoic Acid (RA) in Serum, mES cells undergo preferential neural differentiation and we have observed that they activate a reporter of Wnt signalling (Faunes et al., 2013). This is surprising as Wnt signalling inhibits the neural fate (Aubert et al., 2002; Haegele et al., 2003; Engberg et al., 2010). Analysis of the reporter at the single cell level reveals that not every cell activates Wnt signalling, and therefore raises the possibility that only those cells that are negative for Wnt signalling will develop into neural precursors. If this were the case, there should be a negative correlation between reporter expression and neural differentiation. To test this, we made use of the fact that, in a population of cells self-renewing in Serum and LIF, some cells exhibit low levels of Wnt signalling. Using a fluorescent Wnt reporter, we selected cells in the top 10% of the distribution for analysis of gene expression (Faunes et al., 2013). These cells amount to ∼1% of the population (Fig. 5A) and *Axin2* is only detectable in these cells, in agreement with their high levels of Wnt reporter activity.

Cells that express the Wnt reporter exhibit hallmarks of differentiation (Fig. 5B): *Fgf5* expression and low levels of the pluripotency genes *Rex1*, *Nanog*, *Oct4*, *Sox2*, *Tcf15* and *Otx2* relative to the rest of the population. These cells also express lineage associated genes, but without a bias for particular fates. Thus we observe expression of markers of primitive streak and its derivatives (*T/Bra*, *Mixl1*, *Eomes* and *Gata4*), as expected, and also genes associated with neural development (*Sox1* and *Zfp521*). These results suggest that, as cells exit pluripotency in culture, they activate Wnt signalling and that this activation is not associated with specific lineages, but rather appears to be a feature of the early stages of differentiation. It is known that during self-renewal there is little expression of Wnt genes and that as cells differentiate they express many members of the Wnt family and increase expression of those expressed during self-renewal (Nordin et al., 2008). The increase in reporter expression is likely to be associated with this. The generalized expression of differentiation genes in this population is, at this moment, more difficult to explain.

## Discussion

Here we have analysed gene expression in individual mES cells during self-renewal and the early stages of differentiation. Our results confirm suggestions from population studies that naïve pluripotency (mES cells grown in N2B27 supplemented with BMP4 and LIF) is associated with variable but generally high levels of expression of pluripotency genes (*Nanog*, *Oct4*, *Sox2* and *Rex1*), as well as differentiation markers in a proportion of cells. In ground state pluripotency (2i conditions), these genes are expressed more homogeneously and the expression of differentiation markers is reduced, though we observe a small degree of heterogeneity, which is in agreement with previous observations (Canham et al., 2010) (C. L. Lim, Investigating the dynamics of Nanog heterogeneity in mouse embryonic stem cells, PhD thesis, University of Cambridge, 2011).

When cells exit pluripotency, the expression of the pluripotency markers, with the exception of *Rex1*, does not disappear completely but individual pluripotency genes become restricted to subsets of cells alongside genes affiliated with different lineages: *Oct4* and *Nanog* become associated with the primitive streak gene *T/Bra*, whilst *Sox2* becomes associated with *Sox*1, which marks the neural fate (Wood and Episkopou, 1999; Rivera-Pérez and Magnuson, 2005). Others have reported that individual pluripotency factors are associated with differentiation towards specific lineages (Avilion et al., 2003; Graham et al., 2003; Hart et al., 2004; Osorno et al., 2012). Furthermore, it was recently demonstrated that *Oct4* and *Sox2* specifically inhibit neural and primitive streak differentiation, respectively (Thomson et al., 2011). Our experiments extend these analyses to reveal how dynamic expression of the pluripotency genes during differentiation is used to guide individual cells towards particular lineages. In support of our model, we find that very few cells maintain high-level coexpression of *Nanog*, *Oct4* and *Sox2* during the differentiation process. In this context our experiments reveal that the ability of Wnt/β-catenin signalling to promote Primitive Streak differentiation is dependent on *Nanog* and that this interaction is independent of the roles of Nanog and β-catenin in the maintenance of pluripotency (Silva et al., 2009; Wray et al., 2011; Yi et al., 2011).

In the course of our experiments we were surprised to observe cells primed for endoderm and mesoderm in N2B27, which is commonly used as a base medium to generate neural tissue (Ying et al., 2003b; Engberg et al., 2010; Stavridis et al., 2010). Furthermore, after five days in culture most of the cells can be coaxed towards neural fates if treated with high levels of RA, whilst T/Bra protein is rarely detected under these differentiation conditions (A.M.A., unpublished observations). This suggests that the initial phases of differentiation involve a general priming of multiple lineages at the level of single cells, preceding a process of selection that, in N2B27, results in most cells eventually expressing *Sox1* and undergoing neural commitment and differentiation (Abranches et al., 2009). We also notice a very low proportion of single cells coexpressing markers of different lineages, although the number of cells analyzed in our experiment is not very large. This would suggest that multilineage priming, in the manner that has been shown to occur in hematopoietic precursors (Cross and Enver, 1997; Hu et al., 1997) is a minor event during loss of pluripotency. Instead it appears that even the earliest stages of differentiation of mES cells are mediated by changes in gene expression that are specific to individual lineages.

Our experiments confirm previous observations that differentiation is associated with an increase in Wnt/β-catenin signalling (Faunes et al., 2013). Surprisingly, we observe that this activity is not associated with a particular fate and that cells with high levels of Wnt/β-catenin signalling express genes associated with neural as well as primitive streak fates. It may be that this is part of the ‘priming’ process and that in the same manner that cells are primed for particular fates by lineage specific transcription factors, they are also primed for signalling, i.e. signalling is activated indiscriminately and only later becomes restricted to the cells dependent on it, i.e. those fated to the become the Primitive Streak. Further studies should shed light on the meaning of this observation. We have shown that single cell analysis of the early stages of differentiation of mES cells in culture can reveal features of this process that are masked by population averaging. It will be important to extend these studies to include more genes and to carry out a comparative study of single cells from embryos at the equivalent stages.

## Materials and Methods

### Cell culture and differentiation

E14Tg2A wild-type (129/Ola), *Nanog−/−* (44Cre6) (Chambers et al., 2007) and Wnt reporter (Faunes et al., 2013) mES cells were routinely cultured on gelatin-coated tissue culture plastic in serum-containing medium (Glasgow Minimal Essential Medium (Sigma, G5154), Foetal Bovine Serum (10%), non-essential amino acids 0.1 mM (Life Technologies, 11140-050), GlutaMAX 2 mM (Life Technologies, 35050-038), sodium pyruvate 1 mM (Sigma, S8636), 2-mercaptoethanol 100 nM (Life Technologies, 31350-010)) supplemented with 100 U/mL LIF (Leukaemia Inhibitory Factor – Recombinant human, Department of Biochemistry, University of Cambridge). Prior to initiating experiments, cells were transferred to NDiff N2B27 (StemCells Inc, SCS-SF-NB-02) supplemented with 100 U/mL LIF and 10 ng/mL BMP4 (Department of Biochemistry, University of Cambridge) or 3 µM Chiron (CHIR99021, Division of signal transduction, University of Dundee) and 2 µM PD03 (PD0325901, Division of signal transduction, University of Dundee) and cultured for two passages. Cells were propagated by trypsinisation every two days with a split ratio of approximately 1 in 5. For differentiations, cells were trypsinised and reseeded in N2B27 alone at a density of 10,000 cells/cm^2^.

### Quantitative Polymerase Chain Reaction (qPCR)

RNA was isolated from ∼5×10^5^ trypsinised and pelleted mES cells using the RNeasy Mini kit (Qiagen, 74104) according to the manufacturer's instructions, and resuspended in 30 µL distilled water. RNA was reverse transcribed as follows. RNA samples (1 µg in 38 µL nuclease free water) were combined with 2 µL Oligo-dT anchored primers (Life Technologies, 12577-011) and incubated at 80°C for 2 minutes before transferring immediately to ice for 2 minutes. PCR master mix was then added to each sample: 1.5 µl dNTPs (Life Technologies, 18427-013), 12 µl 5× First Strand buffer (Life Technologies, 18080-400), 3 µl 0.1 M DTT (Life Technologies, D-1532), 1.5 µl RNaseOUT (Life Technologies, 10777-019) and 2 µl Superscript III Reverse Transcriptase (Life Technologies, 18080-400). Thermal cycling was carried out as follows: 25°C 10 minutes, 50°C 30 minutes, 70°C 15 minutes. To remove RNA from the resultant cDNA samples 1 µL RNaseH (Life Technologies, 18021071) was added and samples were incubated at 37°C for 20 minutes and then at 80°C for 20 minutes. Samples were diluted 1 in 10 before being analysed for gene expression using a Rotor-gene Q instrument. Reactions were set up using a Qiagility liquid-handling robot (12.5 µL 2× QuantiFast SYBR Green Master Mix (Qiagen, 204052), 0.5 µL primer mix (50 µM each), 9 µL PCR grade water and 3 µL diluted cDNA) and analysed using the 2-step protocol described in the Quantifast manual (95°C for 5 minutes, then 40 cycles of 95°C for 10 s + 60°C for 30 s, followed by melt curve analysis). Fluorescence curves were quantified using the MAK3 algorithm found in the R package qpcR. Primer sequences are listed in supplementary material Table S1.

### Single cell qPCR

FACS was used to distribute individual mES cells into the wells of a 96-well plate containing lysis buffer (see below). The transcriptomes of isolated cells were then amplified according to a previously published protocol (up to step 30) (Tang et al., 2010). The resultant cDNA samples were purified using a PCR purification kit (Qiagen, 28104) and diluted 1 in 10 in PCR-grade water. Gene expression was measured as described above.

### Flow cytometry/FACS

Cells were trypsinised and dissociated, spun down in a microcentrifuge, resuspended in serum-containing medium, then analysed and/or sorted on a Beckman–Coulter Cytomation MoFlo High Performance Cell Sorter.

## Supplementary Material

Supplementary Material
