# Supplementary material for: Single cell lineage analysis of mouse embryonic stem cells at the exit from pluripotency
Source: Biol Open. 2013 Aug 19;2(10):1049–56. doi: 10.1242/bio.20135934 (PMC3798188; doi:10.1242/bio.20135934)
Supplement: Supplementary Material [file supp_2_10_1049_v2_index.html]

Single cell lineage analysis of mouse embryonic stem cells at the exit from pluripotency — Supplementary Material 

# Single cell lineage analysis of mouse embryonic stem cells at the exit from pluripotency

## bio.20135934 Supplementary Material

**Files in this Data Supplement:**

- Supplementary Material - Jamie Trott and Alfonso Martinez Arias doi: 10.1242/bio.20135934
