# Supplementary material for: Single cell lineage analysis of mouse embryonic stem cells at the exit from pluripotency
Source: Biol Open. 2013 Aug 19;2(10):1049–56. doi: 10.1242/bio.20135934 (PMC3798188; doi:10.1242/bio.20135934)
Supplement: Supplementary Material [file supp_bio.20135934_bio.20135934-s1.pdf]

Biology Open

Supplementary Material

Jamie Trott and Alfonso Martinez Arias **doi: 10.1242/bio.20135934**

Table S1. Primer sequences used for analysis of gene expression by qPCR. All amplicons were within 1 kb of the 3′ end of the corresponding transcript to ensure amplicons were amplified representatively during the initial PCR amplification step. Primers were designed using the primer3 web tool (http://primer3.ut.ee).

Gene	Primer	Sequence
Axin2	5′-primer	ACACATGCAGAAATGGGTCA
	3′-primer	ACGTACGGTGTAGCCTTTGG
T/Bra	5′-primer	CTGGGAGCTCAGTTCTTTCG
	3′-primer	GTCCACGAGGCTATGAGGAG
Eomes	5′-primer	GGCTGAGCCTGAGAGTCAAG
	3′-primer	AGAGCCAGCCCTACAACAAA
Fgf5	5′-primer	GCTCAATGATCAGAAGGAGGA
	3′-primer	TCAGCTGGTCTTGAATGAGG
Gapdh	5′-primer	AACTTTGGCATTGTGGAAGG
	3′-primer	GGATGCAGGGATGATGTTCT
Gata4	5′-primer	CCTAAACCTTACTGGCCGTAGC
	3′-primer	ACAATGTTAACGGGTTGTGGAG
Gata6	5′-primer	TGGGAGCCATTTGGTCTATC
	3′-primer	GACCTCAGATCAGCCACGTT
Mixl1	5′-primer	CAAGCACGCTCACAGTCAAT
	3′-primer	GGAAGCTGTTTCCTGAGCTG
Nanog	5′-primer	TACCTCAGCCTCCAGCAGAT
	3′-primer	GCAATGGATGCTGGGATACT
Oct4	5′-primer	CCAATCAGCTTGGGCTAGAG
	3′-primer	CTGGGAAAGGTGTCCCTGTA
Otx2	5′-primer	CTGGGCTGAACATTCCAGTT
	3′-primer	GTCCATTTCAGGTTGCTGGT
Ppia	5′-primer	TTACCCATCAAACCATTCCTTCTG
	3′-primer	AACCCAAAGAACTTCAGTGAGAGC
Rex1	5′-primer	GCGGTGTGTACTGTGGTGTC
	3′-primer	GACAAGCATGTGCTTCCTCA
Sox1	5′-primer	AGACAGCGTGCCTTTGATTT
	3′-primer	TGGGATAAGACCTGGGTGAG
Sox2	5′-primer	CATGAGAGCAAGTACTGGCAAG
	3′-primer	CCAACGATATCAACCTGCATGG
Tcf15	5′-primer	TGGACAGAGGAGAAGATTCCA
	3′-primer	TAGGCCTGGATGGCTAGATG
Tcf3	5′-primer	ACGGTTCTGGATGAGACAGG
	3′-primer	AAGCAGGGAGCTGTTCAGTG
Zfp521	5′-primer	CCTGGACTGATGGATGTGTG
	3′-primer	CGCCAAGATGTAACAAGCAA
